# Editorial: Genetics and mechanism of ciliopathies

**DOI:** 10.3389/fgene.2022.1067168

**Published:** 2022-10-28

**Authors:** Steven Lim Cho Pei, Brian Hon Yin Chung

**Affiliations:** ^1^ Department of Internal Medicine, School of Medicine, Yale University, New Haven, CT, United States; ^2^ Department of Paediatrics and Adolescent Medicine, School of Clinical Medicine, Li Ka Shing Faculty of Medicine, The University of Hong Kong, Hong Kong, China; ^3^ Hong Kong Genome Institute, Hong Kong, China

**Keywords:** cilia, ciliopathies, primary ciliary dyskinesia, exome sequencing, single nucleotide polymorphisms, dynein

Cilium is a microtubule-based hair-like organelle found on almost every type of cells in human body. These cilia can be motile (as in the cilia on respiratory tract epithelial cells) or non-motile (as in the cilia in nephrons of kidneys and photoreceptors on the retina of eyes). Meanwhile, single cilium (e.g., nephrons) or multiple copies of the cilia (e.g., epithelial cells in respiratory tract) are possible and observed in human cells. Despite of their highly complex structure, ciliary structure is conserved in vertebrate and consist of over 600 proteins (Fliegauf et al., 2007) orchestrating in a coordinated manner for its proper functions in development, signaling, homeostasis and sensory functions. Despite of its complicated structure, various signaling pathways were found to be involved in cilia formation, assembly and growth. For instance, hedgehog signaling (Huangfu et al., 2003), Wnt signaling (Clevers and Nusse, 2012), G-protein-coupled receptor signaling (Mykytyn and Askwith, 2017) etc. Were the well-known pathways involved in ciliopathies (Waters and Beales, 2011; Reiter and Leroux, 2017).

Since cilia are almost ubiquitously presence in all cell types in human body, it is not surprising that ciliopathies, which refers to any disorders that affects the ciliary function, cilia itself or its sub-structures, exhibit a large spectrum of anomalies in multiple organs and systems, for instance, brain, kidneys, liver, bone and eyes etc. Because of the complexity of proteins and signaling pathways involved in ciliopathies, the exact molecular mechanism is yet to be elucidated but it is certain that genetic variation like single nucleotide polymorphisms (SNPs), missense or nonsense mutations on cilia and/or related genes etc. played a major role in the pathogenesis. Thanks to the technology advancement in biotechnology and genetics on genome-editing, as well as the prevalence and readily available of next generation sequencing technique on exome and genome, the manuscripts included in this Research Topic can facilitate researchers to have a deeper understanding on the genetic variations in ciliopathies and provide ways for further verification of these changes with genome-editing and thus, test on the signaling pathways and molecular mechanism.

This Research Topic is dedicated to including works that explore any novel identification or characterization of genes, signaling pathways, transcriptomic analyses, and mechanisms on ciliary gene(s) and/or protein(s) involved in ciliopathies. Moreover, recent advancement in diagnosis of ciliary and/or cilia-related diseases, integrative analysis of publicly available data on ciliary genes and/or proteins, articles that summarize the existing data on ciliary pathologies were also included. A total of 13 manuscripts were included in this Research Topic.

Among these works, a number of novel studies revealing the causes and potential mechanisms of ciliopathies has been included. For instance, four reports described the novel variants identifications contributing to various manifestation of ciliopathies, such as nephronophthisis and kidney failure (by novel deletion of *Glis2*:c.560_574delACCATGTCAACGATT, p. H188_Y192del)) (Al Alawi et al.), asthenoteratozoospermia (by missense variant RSPH4A:c.2T>C, p. (Met1Thr) and nonsense variant *RSPH4A*:c.1774_1775del, p. (Leu592Aspfs*5) (Wang et al.), dynein axonemal assembly factor (DNAAF) -4 [by *DNAAF4*:c.733C>T, p.(Arg245*)] (Guo et al.), Meckel Gruber Syndrome (by splice site variant *RPGRIP1L*:c.776 + 1G > A/IVS6 + 1G > A) (Moreno-Leon et al.). These novel findings expanded the variants spectrum related to ciliopathies and provide some reference for others subjects who share the same variants in future.

While there are another three reports on variants that affect the renal and hepatic fibrocystic disease (by missense variant *TULP3*: c.1144C>T, p. (Arg382Trp)) (Jafari Khamirani et al.), showing skeletal ciliopathies (by missense variant *IFT140* (NM_014,714.4) r.2765_2768del; p. (Tyr923Leufs*28)) (Walczak-Sztulpa et al.) and primary ciliary dyskinesia (PCD) in respiratory cilia [by missense variant *DNAI2*:c.740G>A; p. (Arg247Gln)] (Al-Mutairi et al.). All these case reports in this Research Topic, together with other previously published reports in the literatures, enhance our current understanding on the genetics variants contributed to ciliopathies.

Apart from the variant case reports, another three papers illustrated the vital tasks of cilia proteins in cilia assembly and functions were also included. For instance, Lennon et al. focused on the function of *Dnaaf-4* and *Dnaaf-6* in outer dynein arm (ODA) and a subset of inner dynein arm (IDA) assembly using *drosophila* model. While Schultz et al. studied the role of *CFAP300* in dynein complex transport in motile cilia and in dynein arm assembly. Moreover, Nazlamova et al. showed in the CRISPR mutated *PRPF6* and *PRPF31* cell lines, patient-derived retinal organoid cultures and siRNA-treated retinal organotypic cultures that, mutation in the pre-mRNA splicing factor would resulted in defected microtubule and centrosome, ultimately leading to retinal degeneration. These works shed light on the potential mechanisms and signaling pathways for the pathogenesis of ciliopathies.

Furthermore, two articles in this Research Topic have highlighted the importance of genetic testing in the diagnosis of ciliopathies. The work of Chau et al. has demonstrated that genetic testing is essential in the diagnosis of early-onset bronchiectasis. While the project of Antony et al. indicated the significance of genetic diagnosis in a cohort of 37 cases laterality defect with defected left-right patterning. Their efforts have demonstrated that genetic diagnosis is key for the proper diagnosis of ciliopathies and would provide a valuable reference for the clinician.

In addition, it was delighted to have Rusterholz et al. to contribute an insightful review focusing on Joubert Syndrome, which is a rare cilia-related disorder characterized by cerebellar and brain stem malformation with other renal, retinal and skeletal manifestations. Their work has summarized and featured on what have been learnt in understanding Joubert Syndrome from different zebrafish models in the literatures.

In conclusion, despite many unknown and challenges in delineating the exact cause and molecular mechanism of ciliopathies, these studies in this Research Topic have greatly extended our current understanding on the genetic of ciliopathies and shed light on potential signaling pathway down to molecular level. A better understanding of the genetic and molecular mechanism of the disease is crucial for early diagnosis, appropriate treatment and improving the prognosis of patients, thus, this Research Topic would be valuable for further research and beneficial to the patients in future.
